# Have beliefs in conspiracy theories increased over time?

**DOI:** 10.1371/journal.pone.0270429

**Published:** 2022-07-20

**Authors:** Joseph Uscinski, Adam Enders, Casey Klofstad, Michelle Seelig, Hugo Drochon, Kamal Premaratne, Manohar Murthi

**Affiliations:** 1 Department of Political Science, University of Miami, Coral Gables, FL, United States of America; 2 Department of Political Science, University of Louisville, Louisville, KY, United States of America; 3 Department of Cinema and Interactive Media, University of Miami, Coral Gables, FL, United States of America; 4 School of Politics and International Relations, University of Nottingham, Nottingham, United Kingdom; 5 Department of Electrical and Computer Engineering, University of Miami, Coral Gables, FL, United States of America; Georgia State University, UNITED STATES

## Abstract

The public is convinced that beliefs in conspiracy theories are increasing, and many scholars, journalists, and policymakers agree. Given the associations between conspiracy theories and many non-normative tendencies, lawmakers have called for policies to address these increases. However, little evidence has been provided to demonstrate that beliefs in conspiracy theories have, in fact, increased over time. We address this evidentiary gap. Study 1 investigates change in the proportion of Americans believing 46 conspiracy theories; our observations in some instances span half a century. Study 2 examines change in the proportion of individuals across six European countries believing six conspiracy theories. Study 3 traces beliefs about which groups are conspiring against “us,” while Study 4 tracks generalized conspiracy thinking in the U.S. from 2012 to 2021. In no instance do we observe systematic evidence for an increase in conspiracism, however operationalized. We discuss the theoretical and policy implications of our findings.

## Introduction

An emerging research program focused on conspiracy theories has produced a robust literature on the causes and consequences of beliefs in those theories [1]. Numerous studies find that conspiracy theory beliefs are positively associated with non-normative behaviors, including criminal acts [2], and negatively associated with pro-social behaviors, such as vaccination [3,4]. Recent events, such as the election of Donald Trump, the U.S. Capitol riot, and several conspiracy theory-inspired mass shootings, have prompted widespread concern from scholars, journalists, and the mass public about increases in mass conspiracism.

As for the mass public, 73% of Americans believe that conspiracy theories are currently “out of control” and 59% agree that people are more likely to believe conspiracy theories “compared to 25 years ago” [5,6]. Approximately 77% of Americans believe that social media and the Internet are responsible for these increases [7]. Many scholars agree, and view conspiracy theories as indicative of a modern “crisis,” similarly citing new communication technologies as a primary cause [8,9]. Journalists contend that we are living in the “golden age” of conspiracy theorizing, a “post-truth” era in which conspiracy theories “have never spread this swiftly” nor “lodged this deeply in the American psyche” [10–12]. Similar sentiments are frequently expressed in reporting outside the U.S. as well [13]. Government officials worldwide have responded with policy proposals to address, as one U.S. Congressperson put it, the “stupendous rise in the popularity and prevalence” of conspiracy theories [14]. If beliefs in conspiracy theories are increasing the way popular intuitions, scholars, media narratives, and politicians suggest, there is ample reason for alarm.

Despite a litany of claims from different knowledge producers and consumers, little systematic evidence has been provided to show that beliefs in conspiracy theories have increased over time. While we observe troublingly high percentages of people believing in conspiracy theories on cross-sectional polls and limitless conspiracy theory-related Internet content and social media activity, we argue that these observations cannot, alone, be taken as evidence of increases in conspiracism. Single cross-sectional polls cannot speak to temporal change, social media activity cannot shed light on beliefs prior to the proliferation of Internet access, and Internet content and social media interactions bear only a vague resemblance to mass beliefs [15]. However, looking to repeated opinion polls measuring conspiratorial beliefs over time, especially those taken before social media, does constitute one strategy for assessing change. Unfortunately, such efforts have been quite limited.

This is to the detriment of the study of conspiracism beyond questions about the veracity of the people’s and politicians’ perceptions of change over time. First, studies of conspiracism over time can shed important light on the impact of situational factors and other idiosyncrasies on beliefs, providing researchers with a unique window into the dynamics and antecedents of conspiracy theory beliefs. Second, data such as this can also aid researchers in understanding the comparative dynamics of conspiracism across socio-political contexts, such as types of government. Third, the presence or absence of trends in conspiracism can speak directly to recent concerns about the potential role of social media in fueling conspiracy theory beliefs, which has tangible implications for policy regarding online content moderation and access. More generally, concerns about changes to the socio-political landscape that implicate conspiracy theories––from polarization to democratic backsliding––can only be fully tested with data tracking changes in conspiracy theory beliefs over time.

Given the lack of confirmatory evidence and the stakes involved, it is imperative that the popular narrative—*that beliefs in conspiracy theories have increased over time*—be treated as a falsifiable hypothesis, and then carefully and extensively tested with appropriate over time data measuring beliefs. We therefore, examine time series data of varying lengths from several countries, including a wide variety of conspiracy theory beliefs and other operationalizations of conspiracism. Study 1 investigates over time change in the proportion of Americans believing 46 individual conspiracy theories and four pieces of misinformation, with time spans ranging from seven months (e.g., COVID-19 conspiracy theories) to 55 years (e.g., Kennedy assassination conspiracy theories). Study 2 examines beliefs in six specific conspiracy theories across six European countries between 2016 and 2018. Moving away from beliefs in specific conspiracy theories and toward more general operationalizations of conspiracism in the U.S., Study 3 tracks changes in perceptions about which social and political groups are conspiring, and Study 4 analyzes trends in generalized conspiracy thinking from 2012 to2021. Across all studies, we fail to observe compelling evidence that either specific conspiracy theory beliefs or general forms of conspiracism have increased.

## Ethics

All polls fielded by the authors included only adults 18 years and older. Affirmative consent was provided by respondents by checking a box via computer screen before beginning the survey. Details of institutional review board approval for all author-fielded surveys is included in the SI.

## Study 1: Beliefs in specific conspiracy theories

A *conspiracy theory* is an explanation of past, present, or future events or circumstances that cites as the primary cause a small group of powerful people working in secret, for their own benefit, against the common good, and in a way that undermines bedrock ground rules against the widespread use of force and fraud [16]. Furthermore, conspiracy theories have not been judged as likely accurate by the appropriate epistemological bodies using publicly available evidence [17]. A *conspiracy theory belief* is one’s acceptance that a specific conspiracy theory is (likely) true. Our first investigation utilizes more than 40 conspiracy theories to test the simplest version of the hypothesis that conspiracy theory beliefs are increasing:

H_1_: The proportion of people believing specific conspiracy theories in the U.S. has increased over time.

Because we test this hypothesis using numerous conspiracy theories, each conspiracy theory constitutes a unique test. We would reject the null hypothesis (that the proportions believing specific conspiracy theories in the U.S. have not increased over time) in favor of H_1_ if 1) a majority of the conspiracy theory beliefs we examine show evidence of significant increase from Time_1_ to Time_2_, or, if 2) the increases we observe are larger in magnitude than any decreases we observe. We focus on increases in positive expressions of belief (i.e., professing to agree with, or believe, a conspiracy theory) rather than on changes in levels of disagreement or of being unsure. Our hypothesis, and the popular claims it is based on, speak to *increases in belief*, rather than changes in the intensity of non-belief.

To test H_1_, we re-poll the U.S. public about their beliefs in dozens of conspiracy theories, repeating the exact question wordings used on national polls fielded between 1966 and 2020. While such a research design—comparing previous survey findings to more recent ones—offers a straightforward approach, many specifics about what an appropriate test entails are absent from the popular claims. This lack of specificity has made it difficult to falsify such claims; it also leaves us with little guidance as to the proper length between T_1_ and T_2_, or to which conspiracy theories should be examined. We address these details in turn.

First, whether made by scholars, journalists, or policymakers, claims about increases in conspiracy theory beliefs often lack specificity regarding the rate or absolute levels of change in conspiracy beliefs. Some accounts suggest that increases should be swift, easily detectable, and on-going, with people venturing down “conspiracy-fueled rabbit holes” on social media, thus finding “themselves believing in elaborate conspiracy theories about Bill Gates, 5G wireless technology, vaccines and masks,” and then, “within days, they begin to believe that President Donald Trump is waging a secret war to save trafficked children from a cabal of Satan-worshipping baby eaters” [18]. Other accounts are less specific, suggesting only that we have entered the “golden age” of conspiracy theorizing [19].

Anecdotes aside, one may wonder how far apart T_1_ and T_2_ should be. Do conspiracy beliefs increase linearly or nonlinearly? By the week? The year? Should we expect greater increases over a 50-year period than a single year? Should increases only be found during the social media era? Much like the journalistic accounts, previous scholarship also fails to offer clear guidance on these questions, leaving theorizing about the form that a growth (or decay) function might take as either post-hoc or case specific. We remain agnostic to questions regarding the rate of change, with H_1_ positing only that the *levels of conspiracy theory beliefs at T*_*2*_ *are significantly greater than those at T*_*1*_. Therefore, we test H_1_ comprehensively, intentionally altering assumptions and details. Specifically, we employ 1) comparisons ranging from seven months to 55 years, and 2) conspiracy theories that vary both in age and in the presumed salience of the events and circumstances they address. The conspiracy theories we test range from centuries (e.g., Rothschild family) or decades old (e.g., Pearl Harbor attack), to only a year old (e.g., COVID-19). The length of time between our measurements provides ample potential for growth to be detected, even at the shortest time length (seven months).

Second, claims about increases in conspiracy theory beliefs often fail to specify *which* conspiracy theories they are referring to. Many accounts suggest that beliefs in *all* conspiracy theories are increasing [10], implying that growth should be detectable for any conspiracy theory. Furthermore, popular accounts rarely set limits on their claims by, for example, delineating which, if any, conspiracy theories should *not* be expected to increase in popularity over time. Researchers cannot examine *every* conspiracy theory because the universe of conspiracy theories and versions thereof are constantly expanding and seemingly infinite. Given that an investigation of beliefs in *all* or *most* conspiracy theories is impossible, we test H_1_ using a large number of survey items spanning the five categories of conspiracy theories identified by Brotherton, French, and Pickering [20]: government malfeasance (e.g., government assassinating celebrities), extraterrestrial cover-up (e.g., government hiding alien contact), malevolent global conspiracies (e.g., George Soros controlling the world), personal well-being (e.g., vaccines contain tracking devices), and control of information (e.g., Jeffrey Epstein murdered as part of a cover-up). Because this study is situated in the U.S. where partisan politics often inflames conspiracy theory beliefs [21], we also include conspiracy theories involving partisan actors and issues (e.g., Barack Obama faked his birth certificate). Casting this wide net ensures that our results provide a comprehensive test of H_1_ and generalize to the broader universe of conspiracy theories.

Despite taking these steps, we note three natural limitations to the scope of our study. First, because we are interested in change between T_1_ and T_2_, our analyses are restricted to the universe of conspiracy theories previously included on at least one national survey. Second, our analyses must rely on previous measurements at the time they were taken, and not necessarily when the conspiracy theories were initially developed or when the event or circumstance those conspiracy theories address occurred. Third, an increase in conspiracism over time could result in fewer conspiracy-minded individuals participating in polls, thereby providing a downwardly biased estimate of conspiracy beliefs at T_2_. While Study 4, which examines the predisposition toward conspiracy thinking over time and finds stability over the course of a decade, seems to suggest that this is not an issue, we cannot fully rule out the possibility that it is indeed an issue affecting our results. We begin by testing H_1_ with two critical cases that have recently attracted significant attention: conspiracy theories regarding COVID-19 and QAnon. We then further test H_1_ using 37 other conspiracy theories.

### COVID-19 conspiracy theories

Throughout the pandemic, many scholars and journalists argued that COVID-19 conspiracy theory beliefs were on the rise [22]. For this reason, COVID-19 conspiracy theories represent a critical test of H_1_.

**Fig 1:**
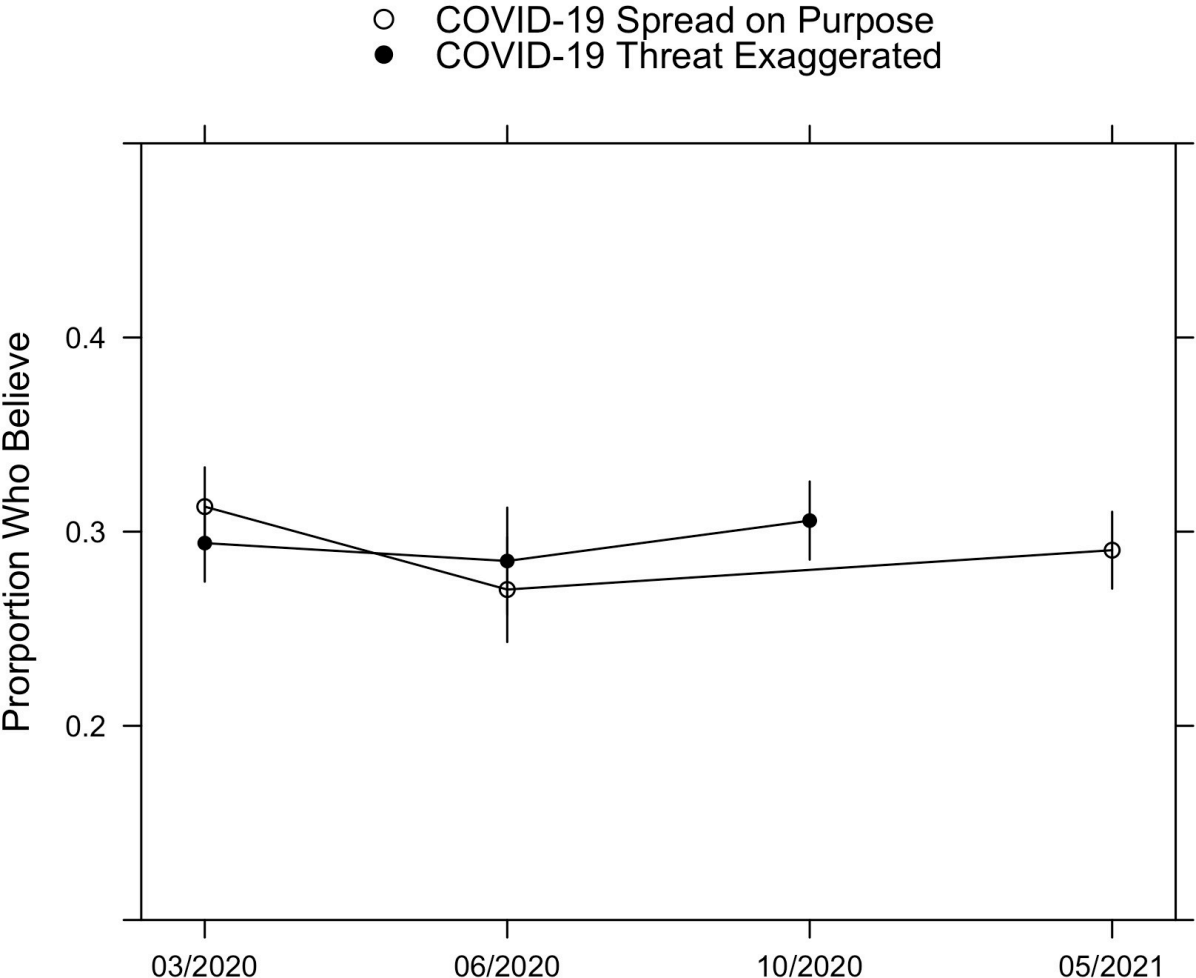
Beliefs in COVID-19 conspiracy theories in the U.S.

We polled on two COVID-19 conspiracy theories in the U.S. in March 2020 (Fig 1): “Coronavirus was purposely created and released by powerful people as part of a conspiracy” and “The threat of coronavirus has been exaggerated by political groups who want to damage President Trump.” Details about survey methodology and sample demographics appear in the appendix. These two theories cover the basic contours of COVID-19 conspiracy theories in the U.S. at the beginning of the pandemic [3]. In repeated polls, however, we observe no increases in either belief (Fig 1). The theory that the coronavirus was “purposely created and released” found support among 31% of Americans in March 2020, 27% in June 2020, and 29% in May 2021. We note that the “lab leak hypothesis” attracted significant attention from policymakers and journalists in the month leading up to our May 2021 survey, yet this does not appear to have fueled increases in the belief that coronavirus was “purposely created and released.” The theory that the coronavirus was exaggerated to “damage President Trump” stood at 29% in March 2020, it was 28% in June 2020, and then increased (non-significantly) to 31% in October 2020 (*p* = 0.086).

Table 1 (below) includes responses to beliefs in three additional pandemic-related conspiracy theories polled in June 2020 and May 2021: “The coronavirus is being used to force a dangerous and unnecessary vaccine on Americans,” “Bill Gates is behind the coronavirus pandemic,” and “The coronavirus is being used to install tracking devices inside our bodies.” All three decreased; the Bill Gates and tracking device theories significantly decreased three and six points, respectively. We observe no evidence of increased beliefs for these five COVID-19 conspiracy theories precisely when increases were most expected. Our results are congruent with those produced by Romer & Jamieson [3], who find that beliefs in three different COVID-19-related conspiracy theories were stable between March and July 2020.

**Table 1:** Change in COVID-19 conspiracy beliefs and misinformation between June 2020 and May 2021.

Question wording	Percent, June 2020	Percent May 2021	*Diff*.	*p*-value for difference
**Conspiracy theories**				
1. The coronavirus is being used to force a dangerous and unnecessary vaccine on Americans.	25	24	−1	0.541
2. Bill Gates is behind the coronavirus pandemic.	13	10	−3	0.012
3. The coronavirus is being used to install tracking devices inside our bodies.	18	12	−6	<0.001
**Misinformation**				
1. The number of deaths related to the coronavirus has been exaggerated.	29	36	+7	<0.001
2. Hydroxychloroquine can prevent or cure COVID-19.	18	18	0	>0.999
3. 5G cell phone technology is responsible for the spread of the coronavirus.	11	7	−4	<0.001
4. Putting disinfectant into your body can prevent or cure COVID-19.	12	6	−6	<0.001

Note: *P*-value corresponds to two-tailed difference in proportions test. All polls are of U.S. adults. Proportions correspond to those saying they “agree” or “strongly agree” with each sentiment. June 2020 and May 2021 polls were both online, opt-in.

We also examine changes in four pieces of COVID-19 misinformation often tied to conspiracy theories (bottom, Table 1). The hydroxychloroquine item showed no change over time, while beliefs regarding 5G and disinfectant decreased significantly (−4 and −6 points, respectively). The only increase identified regarded the idea that the number of COVID-19-related deaths was exaggerated (+7 points). Precisely during the timeframe in which these beliefs were widely said to be spreading, we observe no evidence of over time growth in the five COVID-19 conspiracy theory beliefs we queried; of the four pieces of misinformation we polled on, only one showed evidence of increase.

### QAnon belief and related conspiracy theories

Our second critical case involves QAnon. Numerous reports claimed that belief in QAnon was “spreading,” “growing,” and riding the pandemic “to new heights” [18,23,24]. QAnon adherents believe that a government insider sent them secret messages about President Trump’s battle against the sex-trafficking deep state [25]. Beyond this canonical belief, QAnon supporters believe heterogenous collections of conspiracy theories regarding the deep state, Satanic cults, and sex abuse [26], many of which existed long before QAnon’s emergence in 2017 [27] and find support among a more extensive base than the typically small number of Americans who express belief in QAnon, specifically [28]. We, therefore, gauge belief in QAnon using a variety of survey items in Table 2.

**Table 2:** Change in QAnon and QAnon-related beliefs over time.

Question wording	Time 1	Time 2	Diff.	*p*-value for difference
	Percentage 1	Percentage 2		
1. Are you a believer in QANON? (Yes/No)	5 (08/2019)	6 (05/2021)	+1	0.205
2. There is a “deep state” embedded in the government that operates in secret and without oversight.	43 (03/2020)	44 (05/2021)	+1	0.521
3. Elites, from government and Hollywood, are engaged in a massive child sex trafficking racket.	35 (10/2020)	34 (05/2021)	−1	0.504
4. Jeffrey Epstein, the billionaire accused of running an elite sex trafficking ring, was murdered to cover-up the activities of his criminal network.	50 (03/2020)	48 (05/2021)	−2	0.203
	Average 1	Average 2		
5. QAnon movement (feeling thermometer, 0–100)	21 (07/2019)	16 (05/2021)	−5	<0.001

**Note:** *P*-value corresponds to two-tailed difference in proportions test. All polls are of U.S. adults. Proportions correspond to those saying they “agree” or “strongly agree” with each sentiment. All polls were both online, opt-in.

The first, and most direct, asks “Are you a believer in QANON?”, to which five percent of respondents replied “yes,” in August 2019. We re-polled this question in May 2021, observing a nonsignificant increase of one point. We then examined beliefs in conspiracy theories often associated with QAnon including those regarding the “deep state,” elite sex traffickers, and Jeffrey Epstein. Consistent with other polls [e.g., 26], these theories garner support from between 34 and 50 percent of Americans, reaching far outside the six percent who identify as a “believer in QAnon.” While the baseline levels of belief in these theories are normatively troubling, in no instance do we observe evidence of significant over time increases.

Finally, we asked respondents to rate the “QAnon movement” on a feeling thermometer ranging from 0 (very “cold,” negative feelings) to 100 (very “warm,” positive feelings). If belief in or support for QAnon were increasing, we would expect to observe increases in the average rating assigned to it. However, we find the opposite: in July 2019, the average rating was 21 and by May 2021, the average rating decreased to 16. In total, belief in and support for QAnon, as well as beliefs in ideas that the QAnon movement broadly adopted, remained stable throughout the pandemic and 2020 election cycle, precisely when the QAnon movement was widely perceived to be increasing in size.

### Other conspiracy theories

Moving beyond COVID-19 and QAnon, our analysis continued with a search of the Roper Center for Public Opinion database––the most comprehensive repository of publicly available polling data in the U.S.—for survey items about conspiracy theories that had been administered to national samples in the past. Our search identified only 10 of such items prior to 2010, reflecting the fact that attention to conspiracy theories (especially that involving national polling) is largely concentrated in the last decade. Still, the past polls we identified address a range of topics: the assassinations of President Kennedy, Robert Kennedy, and Martin Luther King Jr., as well as the moon landing, UFOs, AIDS, Pearl Harbor, O.J. Simpson, the Reagan Administration, and the police. We further expanded our search by utilizing items drawn from prominent studies spanning the last decade [29,30].

Our search identified a total of 37 items (polled between 1966–2020) addressing conspiracy theories that vary in who they accuse, which groups in society are/were likely to believe them, the types of events or circumstances they seek to explain, how long they have existed, how widely believed they are/were, and their relative salience. To enable precise comparisons, we re-polled each of the 37 items, retaining the exact question wording and response options used in previous surveys. In all cases, T_2_ polls were conducted online using an opt-in, quota-based sampling procedure; T_1_ polls were sampled using a mixture of random sampling and opt-in procedures and fielded over the telephone (n = 10 with a live interviewer, n = 6 automated), in-person (n = 2), and online (n = 56). We also note that some surveys in the past were restricted to registered voters; in those instances, we also restrict our analysis of the follow-up survey to registered voters. The appendix contains detailed information about all items and polls.

In Table 3, we observe little support for H_1._ Of the 37 conspiracy theory beliefs examined, only six show a significant over time increase, ranging from four to 10 percentage points in magnitude. Of the remaining 31, 16 show no significant change and 15 show a statistically significant decrease. Significant decreases range in magnitude from three to 31 points. The average change across all conspiracy beliefs is −3.84 points. Altogether, the number of conspiracy theory beliefs that increased—six out of 37—is outweighed by the 31 conspiracy theories showing either no change or a significant decrease.

**Table 3:** Change in 37 additional conspiracy beliefs over time.

Question wording	Percentage 1 (Time 1)	Percentage 2 (Time 2)	Diff.	*p*-value for difference
1. Humans have made contact with aliens and this fact has been deliberately hidden from the public.	23 (07/2019)	33 (03/2020)	+10	<0.001
2. Do you think the U.S. government has engaged in the assassination of entertainers who have tried to spread a counterculture message they didn’t like, such as John Lennon, Kurt Cobain, Tupac Shakur, and others, or not?^a^^b^	12 (09/2013)	20 (05/2021)	+8	<0.001
3. Billionaire George Soros is behind a hidden plot to destabilize the American government, take control of the media, and put the world under his control.	19 (10/2011)	26 (05/2021)	+7	<0.001
4. Do you think one man was responsible for the assassination of President Kennedy, or do you think there were others involved?^b^	50 (12/1966)	56 (05/2021)	+6	<0.001
5. Do you believe that the pharmaceutical industry is in league with the medical industry to "invent" new diseases in order to make money, or not? ^a^^b^	15 (03/2013)	20 (05/2021)	+5	<0.001
6. Thinking about space exploration, do you think the government staged and faked the Apollo moon landings, or don’t you feel that way?^b^	6 (07/1995)	10 (05/2021)	+4	<0.001
7. Do you believe media or the government adds secret mind-controlling technology to television broadcast signals, or not? ^a^^b^	15 (03/2013)	17 (05/2021)	+2	0.132
8. Do you believe the government adds fluoride to our water supply, not for dental health reasons, but for other, more sinister reasons, or not? ^a^^b^	9 (03/2013)	11 (05/2021)	+2	0.067
9. Do you think the government is keeping information from the public that shows U.F.O.’s (Unidentified Flying Objects) are real or that aliens have visited the Earth?^b^	49 (06/1996)	50 (05/2021)	+1	0.637
10. Hillary Clinton conspired to provide Russia with access to nuclear materials.	28 (03/2020)	29 (04/2021)	+1	0.481
11. The U.S. government is mandating the switch to compact fluorescent light bulbs because such lights make people more obedient and easier to control.	11 (10/2011)	12 (05/2021)	+1	0.325
12. Health officials know that cell phones cause cancer but are doing nothing to stop it because large corporations won’t let them.	20 (09/2013)	20 (05/2021)	0	>0.999
13. Certain U.S. government officials planned the attacks of September 11, 2001, because they wanted the United States to go to war in the Middle East.	19 (10/2011)	19 (05/2021)	0	>0.999
14. Regardless of who is officially in charge of governments and other organizations, there is a single group of people who secretly control events and rule the world together.	35 (03/2020)	35 (10/2020)	0	>0.999
15. The number of Jews killed by the Nazis during World War II has been exaggerated on purpose.	15 (03/2020)	15 (10/2020)	0	>0.999
16. Climate change is a hoax perpetrated by corrupt scientists and politicians.	19 (07/2019)	19 (10/2020)	0	>0.999
17. Barack Obama faked his citizenship to become president.	20 (03/2020)	19 (05/2021)	−1	0.422
18. Do you completely agree, mostly agree, mostly disagree, or completely disagree that AIDS is a form of systematic destruction of minorities like blacks and Hispanics?^b^	16 (11/1995)	15 (05/2021)	−1	0.416
19. The dangers of vaccines are being hidden by the medical establishment.	30 (03/2020)	29 (05/2021)	−1	0.486
20. The Food and Drug Administration is deliberately preventing the public from getting natural cures for cancer and other diseases because of pressure from drug companies.	37 (09/2013)	35 (05/2021)	−2	0.235
21. The one percent (1%) of the richest people in the U.S. control the government and the economy for their own benefit.	55 (03/2020)	52 (05/2021)	−3	0.056
22. A powerful family, the Rothschilds, through their wealth, controls governments, wars, and many countries’ economies.	29 (03/2020)	26 (05/2021)	−3	0.033
23. The AIDS virus was created and spread around the world on purpose by a secret organization.	22 (03/2020)	19 (06/2020)	−3	0.054
24. The dangers of 5G cellphone technology are being covered up.	26 (03/2020)	23 (10/2020)	−3	0.027
25. Do you feel that the Assassination of Senator Robert Kennedy was the act of one individual or part of a larger conspiracy?^b^	48 (03/1981)	43 (05/2021)	−5	0.009
26. The dangers of genetically-modified foods are being hidden from the public.	45 (03/2020)	40 (05/2021)	−5	0.001
27. School shootings, like those at Sandy Hook, CT and Parkland, FL are false flag attacks perpetrated by the government.	17 (03/2020)	12 (10/2020)	−5	<0.001
28. Do you believe that Osama bin Laden is dead, or do you think he is still alive?^b^	11 (06/2011)	5 (05/2021)	−6	<0.001
29. Donald Trump colluded with Russia to rig the 2016 presidential election.	41 (07/2019)	34 (05/2021)	−7	<0.001
30. Some people are hiding the truth about the December 14, 2012 school shooting at Sandy Hook Elementary in order to advance a political agenda.^a^^b^	25 (04/2013)	16 (05/2021)	−9	<0.001
31. Some people have argued that President Franklin D. Roosevelt knew about Japanese plans to bomb Pearl Harbor but did nothing about it because he wanted an excuse to involve the U.S. (United States) on the side of the allies in the war.^b^	31 (11/1991)	19 (05/2021)	−12	<0.001
32. Republicans won the presidential elections in 2016, 2004, and 2000 by stealing them.	27 (03/2020)	15 (05/2021)	−12	<0.001
33. Do you believe global warming is a hoax, or not?^b^	37 (03/2013)	19 (05/2021)	−18	<0.001
34. Do you think there was a police conspiracy to frame O.J. Simpson or not?^b^	34 (10/1995)	11 (05/2021)	−23	<0.001
35. Do you feel that the Assassination of Martin Luther King was the act of one individual or part of a larger conspiracy?^b^	59 (03/1981)	33 (05/2021)	−26	<0.001
36. Do you think there is, or is not, a national conspiracy to kill police?^b^	44 (11/1970)	16 (05/2021)	−28	<0.001
37. Do you think that the Reagan campaign made a deal with the Iranians to hold the American hostages in Iran until after the 1980 presidential election or not?^b^	43 (07/1991)	12 (05/2021)	−31	<0.001

**Note:** *P*-value corresponds to two-tailed difference in proportions test. All polls are of U.S. adults. Where response options are not dichotomous (e.g., yes/no, believe/don’t believe), the proportion expressing belief is those who “agree” or “strongly agree” with a sentiment.

^a^Registered voters only.

^b^Explicit “don’t know” or “no opinion” option provided; otherwise respondents could opt-out of answering or select “neither agree nor disagree”.

To account for these findings, one might wonder whether the conspiracy theories that fail to show an increase, or that show a decrease, are somehow systematically different from those that show an increase. Examining the beliefs that increased in Table 3 reveals timespans, subject areas, baseline levels of belief, and question wording types similar to those that decreased or remained stable. For example, the largest decrease (31 points) regards beliefs about the 1980 release of the hostages from Iran, which were initially polled in 1991. On the one hand, it could be that this conspiracy theory lost popularity due to its age. But an attempt to discount this item from our analysis for this reason would require significant post-hoc theorizing, especially given that some of the largest increases we observe are among conspiracy theories that also address similarly dated topics (JKF, aliens, John Lennon, and George Soros) that were also initially polled on many years ago. The salience of a conspiracy theory likely has something to do with its age or the age of the object it addresses, but salience is also idiosyncratic and the product of numerous factors. The “fluorescent lightbulb” conspiracy theory (Table 3, #11), which did not increase over time (*p* = 0.325), might shed some light on the potential importance of salience. Because this theory was concocted by researchers [30], it––to our knowledge––never spread among the public and its salience was therefore held constant. Hence, when the salience of, and information about, a conspiracy theory and the topic it addresses are stable over time (there never was any organic public attention paid to this theory), beliefs appear to remain stable over time as well.

That said, we took additional steps to investigate whether there is a relationship between the “age” of a conspiracy theory and belief in it. We first computed the correlation between a) the difference in proportion of believers listed in Table 1 (column 4) and b) the time (in years) between T_1_ and T_2_. The correlation is –0.32 (*p* = 0.010), signifying that the longer a conspiracy theory has been around, the less support it garners. Second, we examined whether there was any relationship between the time a given conspiracy theory belief was first surveyed in our data and the proportion of individuals supporting it at that time––perhaps individual conspiracy theory beliefs are not *increasing* over time, but rather new conspiracy theories are garnering more initial support today than conspiracy theories did in the past. Here, too, we find no support for such a notion; instead, there is a negative correlation (*r* = –0.44, *p* = 0.006) between the date each conspiracy theory was first polled and level of support. Thus, it seems that conspiracy theories tend to lose, rather than gain, believers over time and that “newer” conspiracy theories are not attracting more believers than did conspiracy theories in the past. These observations cut against the popular claims upon which H_1_ is based. That said, we suspect that individual conspiracy theories respond to different forces over time, attracting more or fewer adherents for idiosyncratic reasons, just like many other topics of public opinion do. We also consider the potential role of “don’t know” and “no opinion” responses, especially as they pertain to observed changes in “older” conspiracy theory beliefs; this analysis, which is presented in the appendix, does not reveal an effect in “don’t know” responses.

One might additionally wonder if survey methodologies and modes (e.g., phone versus computerized surveys) are affecting our results. First, we note that our survey samples were constructed to be representative of the U.S. adult population according to the most recent census estimates; this information is presented in the appendix. Second, we suspect that changes in polling methods would work in favor of H_1_, rather than against it, given that surveys guided by a live interviewer––the norm for most polls more than 10 years old––would be more likely to trigger social desirability bias, thereby depressing stated conspiracy beliefs, compared to computerized polls where no interviewer is present. However, we observe no patterns suggesting that our findings are systematically impacted by polling method. Indeed, even when our comparisons employ the same sampling method and administration mode at T_1_ and T_2_, we observe more decreases than increases. In the appendix, we further interrogate the potential impact of survey mode and questionnaire design; in no instance do we observe patterns suggesting that mode or design features are confounding inferences.

Given the frequency of claims about contemporary America entering a “golden age” of conspiracy theories [10,19,31], we expected to find near-universal support for H_1_, regardless of the specific conspiracy theories or the timeframes used in our comparisons. However, our analyses failed to produce consistent evidence of such. Out of 46 total conspiracy theories and related misinformation examined in Study 1, we found only seven with significant increases, but 22 with no significant change and 17 with significant decreases. Moreover, the conspiracy theories that have recently attracted the most concern over their supposed growth (those regarding COVID-19, QAnon, and vaccines) do not appear to be growing in popularity.

## Study 2: Conspiracy theory beliefs across cultures

To examine whether the findings outlined in Study 1 are confined to the U.S., we now test our central hypothesis cross-nationally. In partnership with YouGov, we polled beliefs about six conspiracy theories across six European countries in both 2016 and 2018. Our reconfigured hypothesis is as follows:

H_2_: The proportion of people believing specific conspiracy theories in Germany, Great Britain, Italy, Poland, Portugal, and Sweden has increased over time.

These countries were chosen because they vary in gross domestic product, population, income inequality, political systems, and location within Europe (north/south, east/west), all of which may impact belief in conspiracy theories. We leverage cross-national variability to provide not only more, but potentially different, tests of the central hypothesis.

The surveys utilized here meet the specifications laid out in Study 1, asking conspiracy theory questions a) the same way to b) national samples c) at least twice (in 2016 and again 2018). These surveys also provide an adequate timeframe for detecting growth (two years), as well as a range of conspiracy theories spanning the five categories identified by Brotherton, French, and Pickering [20]. Respondents were asked to select which of the following “statements,” if any, they believe (labels in parentheses):

1. Humans have made contact with aliens and this fact has been deliberately hidden from the public. (*Alien Contact*)
2. The AIDS virus was created and spread around the world on purpose by a secret group or organisation. (*AIDS Virus*)
3. Regardless of who is officially in charge of governments and other organisations, there is a single group of people who secretly control events and rule the world together. (*Control World*)
4. The official account of the Nazi Holocaust is a lie and the number of Jews killed by the Nazis during World War II has been exaggerated on purpose. (*Holocaust Denial*; excluded in German sample)
5. The idea of man-made global warming is a hoax that was invented to deceive people. (*Global Warming*)
6. The Government is deliberately hiding the truth about how many immigrants really live in this country. (*Hide Immigrants*)

**Fig 2:**
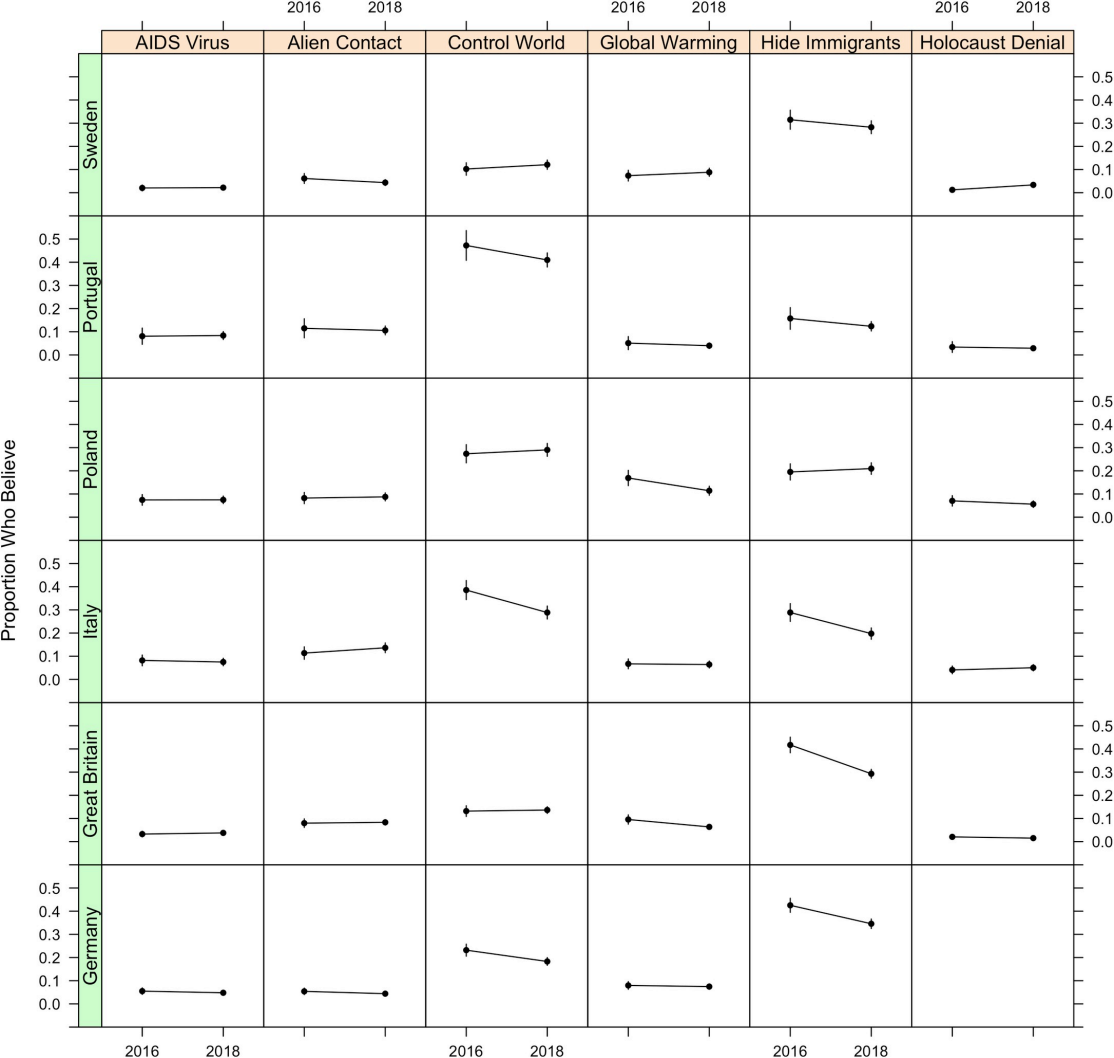
Proportion of adults across 6 European countries who express agreement with each conspiratorial sentiment over time.

Fig 2 shows the proportion of respondents selecting each conspiracy theory, in each country, by year. Out of the 35 comparisons presented, the proportion of believers significantly increased over time in only one: Holocaust denial in Sweden increased from 1% to 3% of the population, (*p* = 0.016). By contrast, we observe significant over time decreases in seven of the comparisons. This leaves 27 of the 35 total cases with no statistically significant change.

These findings align with those of Study 1––most change is not significant and, of the significant changes we observe, decreases outnumber increases. Because we examine only a small number of conspiracy beliefs across six countries, we cannot preclude the possibility of increases in other conspiracy beliefs, or in other countries. Therefore, while we fail to find supportive evidence during a time and in contexts where growth was widely expected [32], we recommend that future studies examine trends in more countries with more conspiracy theories.

## Study 3: Beliefs about which groups are conspiring

In our third study, we transition from beliefs in specific conspiracy theories to perceptions about which groups are likely to be conspirators. Our third hypothesis is as follows:

H_3_: The number of groups Americans believe are conspiring has increased over time.

While we are agnostic as to whether it is normatively worse for people to believe that multiple groups, as opposed to one group, are actively conspiring against them, this constitutes an ancillary test of the general hypothesis: even though the proportion of individuals expressing belief in specific conspiracy theories appears largely stagnant, the number of groups and institutions that people believe are behind those supposed conspiracies could be increasing.

**Fig 3:**
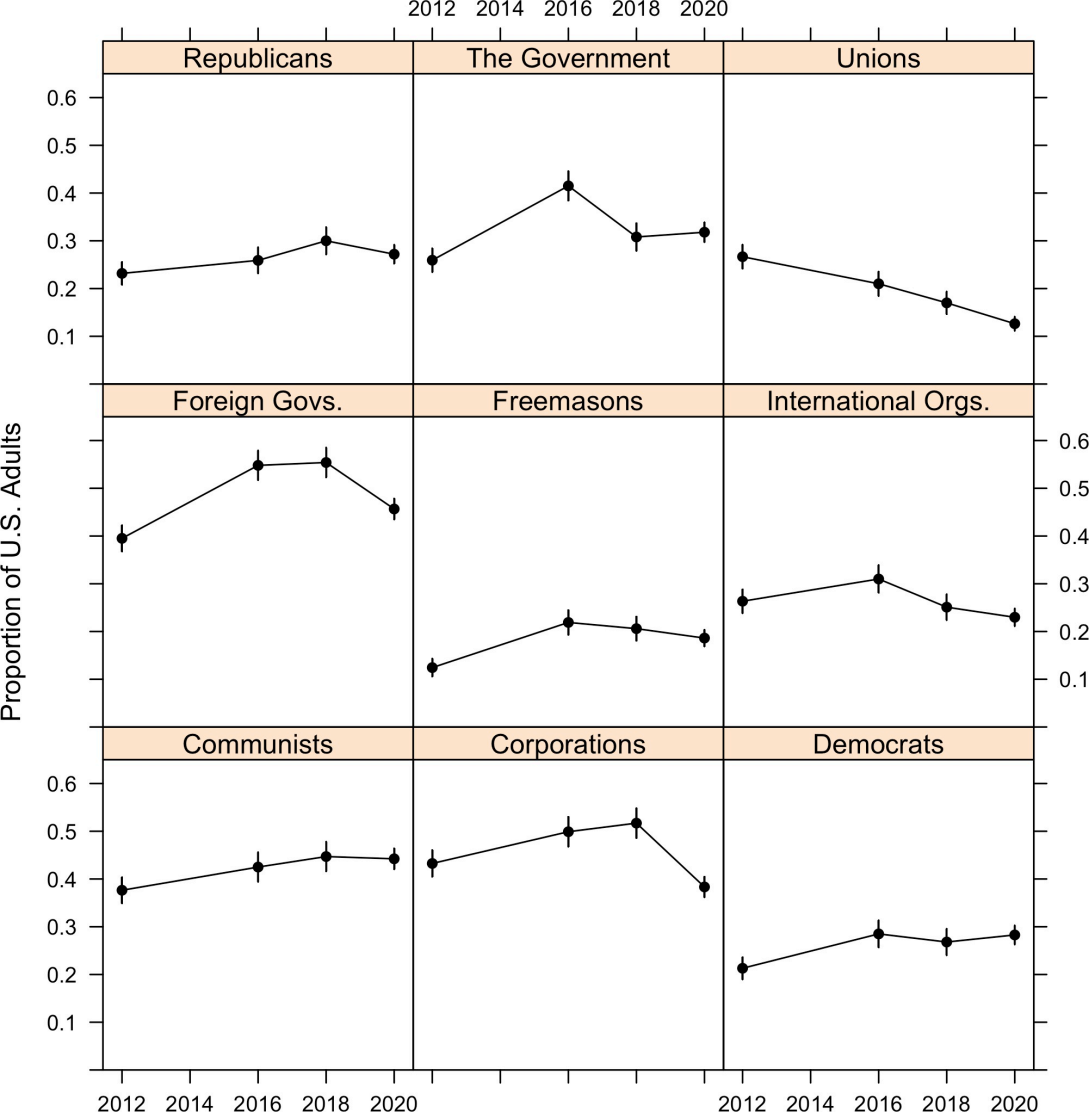
Proportion of U.S. adults who beliefs that each group is “likely to work in secret against the rest of us” over time.

To test this possibility, we asked respondents in four surveys fielded in October 2012, 2016, 2018, and 2020 which of several groups are “likely to work in secret against the rest of us?” Respondents could choose one or more of nine groups: “corporations and the rich,” “Republicans or other conservative groups,” “Democrats or other liberal groups,” “Communists and Socialists,” “the government,” “foreign countries,” “international organizations (e.g., United Nations, International Monetary Fund, World Bank),” “the Freemasons or some other fraternal group,” and “labor unions.” Respondents could also select “none of the above,” or choose not to select any of the options, which we also classified as “none of the above.” Fig 3 plots the proportion of respondents selecting each option, by year/survey.

Across the nine years, we observe average decreases for three groups (corporations, international organizations, and unions) and average increases for the six remaining groups, all of which are significant (*p*<0.05). That said, the decreases are larger than the increases: the average change in proportion across all nine groups of conspirators is –0.13. This is further reflected in an examination of the average count of conspirators that respondents selected: in 2012 respondents selected 2.56 groups, on average, and 2.70 groups in 2020. This difference is not statistically significant at conventional levels (*p* = 0.09). In sum, while fears about particular conspirators ebb and flow over time, we do not observe an average increase in the number of conspirators that people are worried about. Moreover, decreases in the proportion of individuals expressing worry about various conspirators outweigh the observed increases.

## Study 4: Conspiracy thinking

Finally, to answer perhaps the most important question regarding the role of conspiracy theories in contemporary culture—*have people become more conspiracy-minded*, *in general*, *over time*?—we examine temporal trends in the general predisposition to interpret events and circumstances as the product of real conspiracies [20], *conspiracy thinking*. This analysis, like Study 3, is particularly useful because it circumvents the trappings of individual conspiracy theories (e.g., the details, supposed evidence, and salience) that can make generalization difficult. Our fourth hypothesis follows:

H_4_: The average level of conspiracy thinking in the U.S. has increased over time.

We use eight surveys of U.S. adults fielded between October 2012––the earliest known measure of generalized conspiracy thinking on a national survey––and May 2021 to test this hypothesis. Each of our surveys measure conspiracy thinking the same way: the American Conspiracy Thinking Scale (ACTS), first developed by Uscinski and Parent [33] and based on items from McClosky & Chong [34]. This measure of conspiracy thinking has been previously validated in numerous published studies and is strongly associated with beliefs in a wide range of specific conspiracy theories [35–39]. Respondents are asked to react, using five-point scales ranging from “strongly disagree” (1) to “strongly agree” (5), to each of the following four items:

1. Even though we live in a democracy, a few people will always run things anyway.
2. The people who really “run” the country are not known to the voters.
3. Big events like wars, the recent recession, and the outcomes of elections are controlled by small groups of people who are working in secret against the rest of us.
4. Much of our lives are being controlled by plots hatched in secret places.

Responses are averaged into a unidimensional and statistically reliable scale; for example, Cronbach’s alpha ranges from 0.76–0.86 across the eight surveys (see appendix for details). We examined the predictive validity of this measure using our most recent (May 2021) survey: respondents in the upper third of the ACTS express belief in more than three times as many specific conspiracy theories than those falling in the lower third of the scale (14 conspiracy theories versus four, respectively), even controlling for attitudinal and demographic factors (see appendix). Predictive validity aside, we do not claim that conspiracy thinking, as measured here, is the causal explanation for conspiracy theory beliefs; instead, we merely propose that this measure is a proxy for the general propensity to believe conspiracy theories.

**Fig 4:**
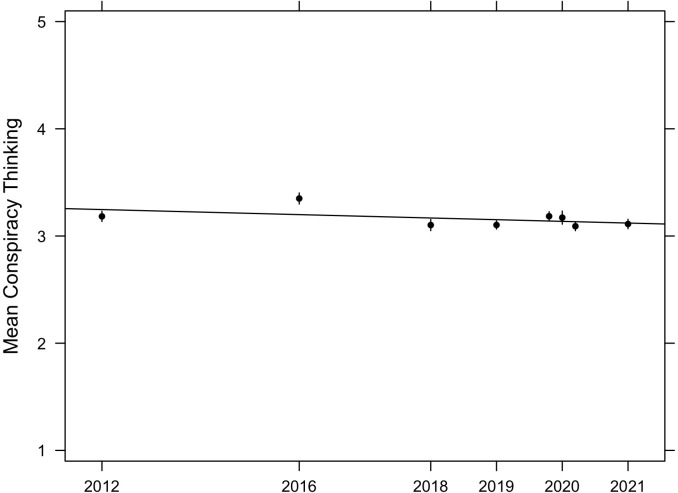
Average level of conspiracy thinking. Conspiracy thinking is measured with the ACTS (range 1–5), over time. Vertical lines are 95% confidence intervals, horizontal line is OLS fit.

Fig 4 plots the average of the ACTS for each survey, along with 95% confidence intervals and an OLS fit line (*β* = –0.45, *p* = 0.269) to highlight trends (or the lack thereof). While we observe an increase moving from 2012 to 2016 (*p*<0.001), it is substantively small at only 0.17 units on a five-point scale. All other time points display averages that are statistically indistinguishable from 2012 levels (March 2020, *p* = 0.966; June 2020, *p* = 0.770) or statistically lower than 2012 levels (2018, *p* = 0.026; 2019, *p* = 0.011; October 2020, *p* = 0.006; May 2021, *p* = 0.039). In short, we observe no average increase in conspiracy thinking over time. This lack of an upward trend is disconfirmatory of the central hypothesis that conspiracism has increased over time. We note, however, that average levels of conspiracy thinking are potentially concerning, even if they are not increasing. Therefore, researchers should continue investigating the scope of conspiracy thinking and develop strategies for addressing it.

## Conclusion

Numerous cross-sectional polls show that large numbers of people believe conspiracy theories, and online conspiracy theory content is plentiful. Perhaps because of this, many scholars, journalists, and policymakers are concerned that conspiracism is increasing. However, little *systematic* evidence demonstrating such increases has been produced. As one journalist at *Vox* put it, “there’s no hard evidence that conspiracy theories are circulating more widely today than ever before. But…it has certainly seemed like average Americans have bought into them more and more” [40].

The lack of systematic evidence owes to the fact that conspiracy theories became the subject of a sustained research program only around 2010. Regardless, claims about increases in conspiracy theory beliefs must be both testable and falsifiable if they are to be taken seriously. Minimally, hypothesized increases should be detectable using standard methods (such as, but not limited to, polling). If such hypotheses cannot be substantiated with supportive evidence, they should be appropriately qualified, refined to match the available evidence, or abandoned.

Across four studies––including four distinct operationalizations of conspiracism, temporal comparisons spanning between seven months and 55 years, and tens of thousands of observations from seven nations––we find only scant evidence that conspiracism, however operationalized, has increased. Although beliefs in 13 out of 52 conspiracy theories significantly increased over time (including those in both Study 1 and Study 2), these increases do not constitute sufficient evidence against the null hypothesis. In fact, we identified more decreases than increases, and the decreases were larger in magnitude than the increases. That only a quarter of the conspiracy theories we examined found more support over time––none of which involve the COVID-19 pandemic or QAnon––contradicts common wisdom.

The baseline levels of conspiracism we observe are concerning and social scientists should continue efforts at correcting them [e.g., 41]. By the same token, our finding that conspiracy theory beliefs are generally not increasing has implications for public discourse. Claims that beliefs in conspiracy theories are on the rise suggest that a new factor is to blame, or that a meaningful change in an old factor has occurred. In this vein, social media has––perhaps erroneously––taken much of the blame for supposed increases in conspiracy theory beliefs [CBS 7], which has implications for policies regarding content moderation and access.

However, we do not observe supporting evidence that beliefs in conspiracy theories or generalized conspiracy thinking have increased during the Internet/social media era. Instead, our findings comport with arguments that the Internet may be less hospitable to conspiracy theories than is often assumed [42]. Our findings also comport with studies demonstrating that online conspiracy theories, “infodemics,” and echo chambers may not be as widespread [43–45] or influential as sometimes claimed [46], and are reflective of studies arguing that people are not engaging with or sharing conspiracy theories online as much as sometimes assumed [47–49]. Finally, the patterns we observe align with a broad literature on conspiracy theory beliefs showing that people are unlikely to believe a conspiracy theory unless they are both 1) already disposed to believe conspiracy theories generally, and 2) inclined towards the content of that particular conspiracy theory or the source from which it emanates [39,50,51]. In other words, online conspiracy theories might not *persuade* as much as *reinforce* existing views. Our findings are more congruent with the latter process than the former.

That said, our investigation is not without limitations. We are limited to the conspiracy theories polled on previously, and we cannot make claims about conspiracy theories we did not investigate. Still, we expect that we are more likely to observe growth in the types of ideas that researchers thought worthwhile to ask the public about than those they chose to ignore. We acknowledge that the many claims about increases in conspiracism are often vague and could mean numerous things. We have therefore tested several operationalizations of conspiracism in our four studies, but future research should continue testing for increases in other ways as well. We further acknowledge that no single study can poll in all political contexts. Some beliefs not included in Study 2 could be increasing in the six European countries polled; moreover, conspiracy theory beliefs could be increasing in some countries not accounted for here. We note that most polling of conspiracy theory beliefs has taken place in the U.S. during the last decade––efforts to comprehensively measure conspiracy theory beliefs with national polls across the globe are only slowly emerging [e.g., 52]. More work outside the U.S. is needed to test our central hypothesis more comprehensively.

We implore caution in making sweeping inferences from our findings. Our study should not be used to make claims about, or to excuse the behavior of, political elites who weaponize conspiracy theories. Moreover, trends in the coverage of conspiracy theories by news outlets or in the rhetorical use of conspiracy theories by political elites fall outside the purview of our investigation, as do the use of conspiracy theories by fake news purveyors, though we recommend that researchers continue to consider these topics.

Questions regarding the growth in conspiracy theory beliefs are important, with far-reaching normative and empirical implications for our understanding of political culture, free speech, Internet regulation, and radicalization. That we observe little supportive evidence for such growth, however operationalized, should give scholars, journalists, and policymakers pause. This is not to dismiss the availability of conspiracy theories online, the large numbers of people who believe in some conspiracy theories, or the potential consequences of those beliefs; nor is it to preclude the possibility of increases in the future, in ways not tested here, or in other socio-political contexts. It may be that conspiracy theories have been a constant, but that scholars, policymakers, and journalists are only recently beginning to pay appropriate attention to them. Thus, our findings offer both good and bad news: good, in that conspiracy theory beliefs are not increasing across the board; bad, in that conspiracy theories may be a more persistent and ubiquitous feature of human society than is desirable. Scholars still have much to discover about the psychology of conspiracy theory beliefs, as well as the role that elite communication and the information environment play in promoting those beliefs. In the meantime, we recommend caution in sounding alarms regarding the “golden age” of conspiracy theories and the degeneration of society into a “post-truth” era.

## Supporting information

S1 File This file contains supplementary information about all polls and analyses. (PDF)
