# Supplementary material for: Have beliefs in conspiracy theories increased over time?
Source: PLoS One. 2022 Jul 20;17(7):e0270429. doi: 10.1371/journal.pone.0270429 (PMC9299316; doi:10.1371/journal.pone.0270429)

Supplemental Appendix:

"Have Beliefs in Conspiracy Theories Increased Over Time?"

Table of Contents

I. Details about author-fielded surveys, page 2–4

II. Details about surveys not fielded by authors, pages 5–7

III. Table with all conspiracy theories in Study 1, pages 8–10

IV. Examining the potential impact of survey design, pages 11–12

V. Examining changes in and impact of "don't know" responses, page 13-14

VI. Psychometric properties of the ACTS, page 15

VII. Predictive validity of the ACTS, pages 16–18

I. Details about author-fielded surveys

Table A1: Original U.S. survey data fielded by authors and used across studies.

**Polling Organization**	**Dates Fielded**	**Sample Size**	**Sample/Sampling Procedure**	**IRB Protocol # (University of [redacted])**
1. CCES	October 2012	1,230	Opt-in YouGov panelists; weighted to be representative	20120757
2. CCES	October 2016	1,000	Opt-in YouGov panelists; weighted to be representative	20120757/ MOD00013692
3. CCES	October 2018	1,000	Opt-in YouGov panelists; weighted to be representative	20120757/ MOD00023764
4. Qualtrics	July 2019	2,000	Quota sample; stratified to be representative	20190623
5. Qualtrics	March 2020	2,023	Quota sample; stratified to be representative	20200095
6. Qualtrics	June 2020	1,040	Quota sample; stratified to be representative	20200673
7. Qualtrics	October 2020	2,015	Quota sample; stratified to be representative	20201154
8. Qualtrics	May 2021	2,021	Quota sample; stratified to be representative	20210244

Note: "CCES"=Cooperative Congressional Election Study; all surveys fielded on U.S. adults (18+).

Table A2: Sociodemographic information about all original U.S. samples, compared to 2010 U.S. Census estimates.

Characteristic	2010 Census Estimate	Qualtrics May 2021	Qualtrics October 2020	Qualtrics June 2020	Qualtrics March 2020	Qualtrics July 2019	CCES Oct. 2018	CCES Oct. 2016	CCES Oct. 2012
Age	38	48	43	46.5	39	50	48	48	47
High school degree	88	97	97	98	95	99	91	89	89
Some college or more	59	71	76	76	60	60	63	59	59
Female	51	50	51	51	52	52	52	52	52
White	72	62	68	60	65	62	70	73	73
Black	13	14	14	17	15	14	13	12	12
Hispanic	16	16	17	27	18	16	9	6	8
n		2,021	2,015	1,040	2,023	2,000	1,000	1,000	1,230

Note: All entries are percentages except age, which is the median.

While median age is slightly higher for our surveys than the 2010 Census estimates, recall that we are able to poll on only those age 18 years old and older, whereas the Census provides the median age of all Americans.

Table A3: Descriptive characteristics of European samples in 2016, by country.

Variable (range)	GB	DE	SE	PT	IT	PL
Trust in Media (0–3)	0.87	1.24	1.25	1.18	1.03	1.06
	(0.73)	(0.81)	(0.92)	(0.72)	(0.76)	(0.73)
Trust Government (0–3)	0.77	0.99	1.13	0.70	0.63	0.56
	(0.74)	(0.81)	(0.94)	(0.65)	(0.72)	(0.71)
Ideology (1–5)	2.86	2.86	3.28	2.71	2.75	3.08
	(1.20)	(0.99)	(1.31)	(1.41)	(1.30)	(1.27)
Religious Thinking (0–3)	0.73	0.92	0.60	1.12	1.25	1.23
	(0.90)	(1.01)	(0.80)	(1.01)	(0.98)	(1.10)
Education (1–5)	2.94	3.02	3.00	3.14	2.88	3.52
	(1.13)	(1.06)	(1.14)	(1.15)	(1.37)	(1.34)
Unemployed (prop.)	0.06	0.04	0.04	0.11	0.13	0.06
	(0.24)	(0.19)	(0.19)	(0.32)	(0.34)	(0.24)
Age (18–87)	48.91	48.42	48.86	43.67	45.96	44.37
	(16.98)	(14.85)	(17.90)	(13.88)	(14.86)	(14.86)
Female (prop.)	0.56	0.50	0.51	0.50	0.52	0.51
	(0.50)	(0.50)	(0.50)	(0.50)	(0.50)	(0.50)
n	1,742	2,039	1,007	500	1,001	1,000

Note: Entries are means (unless where otherwise noted) with standard deviations in parentheses. Country codes are as follows: Great Britain (GB), Germany (DE), Sweden (SE), Portugal (PT), Italy (IT), Poland (PT).

II. Details about surveys not fielded by authors

Table A4: Details about surveys from which the first time points in Table 3 were taken. Only includes those surveys not fielded by the authors (which are described above).

Question wording (response options)	Dates Fielded	Polling Organization	Target	Sample Size
1. Do you believe the government adds fluoride to our water supply, not for dental health reasons, but for other, more sinister reasons, or not? (Do/do not)	3/27/2013–3/30/2013	Public Policy Polling	Registered voters	1,247
2. Do you believe global warming is a hoax, or not? (Do/do not)	3/27/2013–3/30/2013	Public Policy Polling	Registered voters	1,247
3. Do you think the government is keeping information from the public that shows U.F.O.'s (Unidentified Flying Objects) are real or that aliens have visited the Earth? (Yes/no)	6/27/1996–6/28/1996	PSRA/Newsweek Poll	Adults	769
4. Do you believe media or the government adds secret mind-controlling technology to television broadcast signals, or not? (Do/do not)	3/27/2013–3/30/2013	Public Policy Polling	Registered voters	1,247
5. Do you completely agree, mostly agree, mostly disagree, or completely disagree that AIDS is a form of systematic destruction of minorities like blacks and Hispanics?	11/27/1995–12/17/1995	Kaiser Survey on Americans and AIDS/HIV	Adults	1,511
6. Do you think that the Reagan campaign made a deal with the Iranians to hold the American hostages in Iran until after the 1980 presidential election or not?	7/17/1991–7/18/1991	Time/CNN/Yankelovich Clancy Shulman Poll	Adults	1,000
7. Do you think one man was responsible for the assassination of President Kennedy, or do you think there were others involved? (Others/one man)	12/8/1966–12/13/1966	Gallup (AIPO)	Adults	1,469
8. Thinking about space exploration, do you think the government staged and faked the Apollo moon landings, or don't you feel that way? (Yes, staged/no, not staged)	7/19/1995–7/20/1995	Time/CNN/Yankelovich Partners Poll	Adults	1,000
9. Do you believe that Osama bin Laden is dead, or do you think he is still alive? (Alive/dead)	6/3/2011–6/7/2011	CNN/Opinion Research Corporation Poll	Adults	1,015

10. Do you believe that the pharmaceutical industry is in league with the medical industry to "invent" new diseases in order to make money, or not? (Do/do not)	3/27/2013–3/30/2013	Public Policy Polling	Registered voters	1,247
11. Some people have argued that President Franklin D. Roosevelt knew about Japanese plans to bomb Pearl Harbor but did nothing about it because he wanted an excuse to involve the U.S. (United States) on the side of the allies in the war. From what you know or have read, do you agree or disagree with this point of view? (Agree/disagree)	11/21/1991–11/24/1991	Gallup	Adults	1,005
12. Some people are hiding the truth about the December 14, 2012 school shooting at Sandy Hook Elementary in order to advance a political agenda (Agree/disagree/neither agree nor disagree)	4/27/2013–4/29/2013	Farleigh Dickinson University's Public Mind Poll	Registered voters	863
13. Do you think the U.S. government has engaged in the assassination of entertainers who have tried to spread a counterculture message they didn't like, such as JohnLennon, Kurt Cobain, Tupac Shakur, and others, or not? (Yes/no)	9/25/2013–9/26/2013	Public Policy Polling	Registered voters	790
14. Do you feel that the Assassination of Martin Luther King was the act of one individual or part of a larger conspiracy? (One individual/larger conspiracy)	3/30/1981–3/31/1981	Louis Harris & Associates	Adults	1,001
15. Do you feel that the Assassination of Senator Robert Kennedy was the act of one individual or part of a larger conspiracy? (One individual/larger conspiracy)	3/30/1981–3/31/1981	Louis Harris & Associates	Adults	1,001
16. Are you a believer in QANON? (Yes/no)	8/24/2019–8/26/2019	Emerson Polling	Registered voters	1,458
17. The Food and Drug Administration is deliberately preventing the public from getting natural cures for cancer and other diseases because of pressure from drug companies. (SA–SD)	09/2013	YouGov/Eric Oliver	Adults	1,351
18. Health officials know that cell phones cause cancer but are doing nothing to stop it because large corporations won't let them. (SA–SD)	09/2013	YouGov/Eric Oliver	Adults	1,351

19. Certain U.S. government officials planned the attacks of September 11, 2001, because they wanted the United States to go to war in the Middle East. (SA–SD)	10/2011	CCES/YouGov	Adults	1,935
20. The U.S. government is mandating the switch to compact fluorescent light bulbs because such lights make people more obedient and easier to control. (SA–SD)	10/2011	CCES/YouGov	Adults	1,935
21. Billionaire George Soros is behind a hidden plot to destabilize the American government, take control of the media, and put the world under his control. (SA–SD)	10/2011	CCES/YouGov	Adults	1,935
22. Do you think there was a police conspiracy to frame O.J. Simpson or not? (Yes/no)	10/4/1995–10/6/1995	Washington Post	Adults	684
23. Do you think there is, or is not, a national conspiracy to kill policemen? (Is/is not)	10/1970	Gallup/Newsweek	Adults	519

Note: All polls are of Americans. “SA–SD” corresponds to five-point Likert type response options ranging from strongly agree to strong disagree.

III. Table with all conspiracy theories in Study 1

Table A5: Change in 51 conspiracy beliefs over time.

Question wording	Percentage 1 (Time 1)	Percentage 2 (Time 2)	Diff.	*p*-value for difference
1. Humans have made contact with aliens and this fact has been deliberately hidden from the public.	23 (07/2019)	33 (03/2020)	+10	<0.001
2. Do you think the U.S. government has engaged in the assassination of entertainers who have tried to spread a counterculture message they didn't like, such as John Lennon, Kurt Cobain, Tupac Shakur, and others, or not?*	12 (09/2013)	20 (05/2021)	+8	<0.001
3. Billionaire George Soros is behind a hidden plot to destabilize the American government, take control of the media, and put the world under his control.	19 (10/2011)	26 (05/2021)	+7	<0.001
4. The number of deaths related to the coronavirus has been exaggerated.	29 (06/2020)	36 (05/2021)	+7	<0.001
5. Do you think one man was responsible for the assassination of President Kennedy, or do you think there were others involved?	50 (12/1966)	56 (05/2021)	+6	<0.001
6. Do you believe that the pharmaceutical industry is in league with the medical industry to "invent" new diseases in order to make money, or not?*	15 (03/2013)	20 (05/2021)	+5	<0.001
7. Thinking about space exploration, do you think the government staged and faked the Apollo moon landings, or don't you feel that way?	6 (07/1995)	10 (05/2021)	+4	<0.001
8. Do you believe media or the government adds secret mind-controlling technology to television broadcast signals, or not?*	15 (03/2013)	17 (05/2021)	+2	0.132
9. Do you believe the government adds fluoride to our water supply, not for dental health reasons, but for other, more sinister reasons, or not?*	9 (03/2013)	11 (05/2021)	+2	0.067
10. Do you think the government is keeping information from the public that shows U.F.O.'s (Unidentified Flying Objects) are real or that aliens have visited the Earth?	49 (06/1996)	50 (05/2021)	+1	0.637
11. Are you a believer in QANON?*	5 (08/2019)	6 (05/2021)	+1	0.205
12. There is a "deep state" embedded in the government that operates in secret and without oversight.	43 (03/2020)	44 (05/2021)	+1	0.521
13. Hillary Clinton conspired to provide Russia with access to nuclear materials.	28 (03/2020)	29 (04/2021)	+1	0.481
14. The U.S. government is mandating the switch to compact fluorescent light bulbs because such lights make people more obedient and easier to control.	11 (10/2011)	12 (05/2021)	+1	0.325

15. The threat of coronavirus has been exaggerated by political groups who want to damage President Trump.	30 (03/2020)	31 (10/2020)	+1	0.490
16. Hydroxychloroquine can prevent or cure COVID-19.	18 (06/2020)	18 (05/2021)	0	>0.999
17. Health officials know that cell phones cause cancer but are doing nothing to stop it because large corporations won't let them.	20 (09/2013)	20 (05/2021)	0	>0.999
18. Certain U.S. government officials planned the attacks of September 11, 2001, because they wanted the United States to go to war in the Middle East.	19 (10/2011)	19 (05/2021)	0	>0.999
19. Regardless of who is officially in charge of governments and other organizations, there is a single group of people who secretly control events and rule the world together.	35 (03/2020)	35 (10/2020)	0	>0.999
20. The number of Jews killed by the Nazis during World War II has been exaggerated on purpose.	15 (03/2020)	15 (10/2020)	0	>0.999
21. Climate change is a hoax perpetrated by corrupt scientists and politicians.	19 (07/2019)	19 (10/2020)	0	>0.999
22. Barack Obama faked his citizenship to become president.	20 (03/2020)	19 (05/2021)	–1	0.422
23. Do you completely agree, mostly agree, mostly disagree, or completely disagree that AIDS is a form of systematic destruction of minorities like blacks and Hispanics?	16 (11/1995)	15 (05/2021)	–1	0.416
24. The coronavirus is being used to force a dangerous and unnecessary vaccine on Americans.	25 (06/2020)	24 (05/2021)	–1	0.541
25. The dangers of vaccines are being hidden by the medical establishment	30 (03/2020)	29 (05/2021)	–1	0.486
26. Elites, from government and Hollywood, are engaged in a massive child sex trafficking racket.	35 (10/2020)	34 (05/2021)	–1	0.504
27. Coronavirus was purposely created and released by powerful people as part of a conspiracy.	31 (03/2020)	29 (05/2021)	–2	0.165
28. Jeffrey Epstein, the billionaire accused of running an elite sex trafficking ring, was murdered to cover-up the activities of his criminal network.	50 (03/2020)	48 (05/2021)	–2	0.203
29. The Food and Drug Administration is deliberately preventing the public from getting natural cures for cancer and other diseases because of pressure from drug companies.	37 (09/2013)	35 (05/2021)	–2	0.235
30. The one percent (1%) of the richest people in the U.S. control the government and the economy for their own benefit.	55 (03/2020)	52 (05/2021)	–3	0.056
31. A powerful family, the Rothschilds, through their wealth, controls governments, wars, and many countries' economies.	29 (03/2020)	26 (05/2021)	–3	0.033

32. Bill Gates is behind the coronavirus pandemic.	13 (06/2020)	10 (05/2021)	–3	0.012
33. The AIDS virus was created and spread around the world on purpose by a secret organization.	22 (03/2020)	19 (06/2020)	–3	0.054
34. The dangers of 5G cellphone technology are being covered up.	26 (03/2020)	23 (10/2020)	–3	0.027
35. 5G cell phone technology is responsible for the spread of the coronavirus.	11 (06/2020)	7 (05/2021)	–4	<0.001
36. Do you feel that the Assassination of Senator Robert Kennedy was the act of one individual or part of a larger conspiracy?	48 (03/1981)	43 (05/2021)	–5	0.009
37. The dangers of genetically-modified foods are being hidden from the public.	45 (03/2020)	40 (05/2021)	–5	0.001
38. School shootings, like those at Sandy Hook, CT and Parkland, FL are false flag attacks perpetrated by the government.	17 (03/202)	12 (10/2020)	–5	<0.001
39. Do you believe that Osama bin Laden is dead, or do you think he is still alive?	11 (06/2011)	5 (05/2021)	–6	<0.001
40. The coronavirus is being used to install tracking devices inside our bodies.	18 (06/2020)	12 (05/2021)	–6	<0.001
41. Putting disinfectant into your body can prevent or cure COVID-19.	12 (06/2020)	6 (05/2021)	–6	<0.001
42. Donald Trump colluded with Russia to rig the 2016 presidential election.	41 (07/2019)	34 (05/2021)	–7	<0.001
43. Some people are hiding the truth about the December 14, 2012 school shooting at Sandy Hook Elementary in order to advance a political agenda.*	25 (04/2013)	16 (05/2021)	–9	
44. Some people have argued that President Franklin D. Roosevelt knew about Japanese plans to bomb Pearl Harbor but did nothing about it because he wanted an excuse to involve the U.S. (United States) on the side of the allies in the war.	31 (11/1991)	19 (05/2021)	–12	<0.001
45. Republicans won the presidential elections in 2016, 2004, and 2000 by stealing them.	27 (03/2020)	15 (05/2021)	–12	<0.001
46. Do you believe global warming is a hoax, or not?	37 (03/2013)	19 (05/2021)	–18	<0.001
47. Do you think there was a police conspiracy to frame O.J. Simpson or not?	56 (10/2018)	32 (05/2021)	–24	<0.001
48. Do you feel that the Assassination of Martin Luther King was the act of one individual or part of a larger conspiracy?	59 (03/1981)	33 (05/2021)	–26	<0.001
49. Do you think there is, or is not, a national conspiracy to kill police?	44 (11/1970)	16 (05/2021)	–28	<0.001
50. Do you think that the Reagan campaign made a deal with the Iranians to hold the American hostages in Iran until after the 1980 presidential election or not?	43 (07/1991)	12 (05/2021)	–31	<0.001

Note: *P*-value corresponds to two-tailed difference in proportions test. All polls are of U.S. adults. Where response options are not dichotomous (e.g., yes/no, believe/don't believe), the proportion expressing belief is those who "agree" or "strongly agree" with a sentiment. *registered voters only.

IV. Examining the potential impact of survey design

We undertook several analyses to ensure that various elements of survey design were not systematically impacting results in Study 1. Note, however, that Studies 2, 3, and 4 all use data collected via identical methods across data points, so survey design has no bearing on our findings of stability.

First, we investigated whether there are systematic differences in support for conspiracy belief by survey mode. There are four different modes represent in our dataset. Average support for conspiracy theories, by mode, are as follows:

- Online: 25.34 (n=84)
- Automated telephone: 15.50 (n=6)
- Telephone: 33.40 (n=10)
- Face-to-face: 47 (n=2)

Contrary to what one may expect, we find higher levels of reported conspiracy beliefs among those who were interviewed by a trained interviewer, either over the telephone or face-to-face, compared to those who completed their questionnaire online or by automated telephone poll. Because sample sizes are quite small for some modes, we tested the effect of mode in a regression context, including controls for year (so as not to confound the effect of mode and year, which are related) and a dummy variable that simply captures whether the survey was fielded by Qualtrics. Results appear in the first column of Table A6; online is the omitted reference category for the mode variable and standard errors are clustered by conspiracy theory question (of which there are two entries for each question).

None of the three variables we consider are statistically significant. If we remove the Qualtrics dummy variable, we do find a significant (p=0.004) coefficient on the automated telephone category of -14.385, suggesting that automated phone polls showed lower levels of conspiracy theory belief than online polls, on average. Note, however, that there is only one automated phone poll in our dataset—Public Policy Polling—which contains six conspiracy belief questions. Also note that this finding works *against* our principle finding of stability, since automated phone polls are always T_1 and never T_2 (i.e., this pattern is much more likely to result in an observed increase in conspiracy beliefs than a decrease).

Next, we considered whether the number of conspiracy belief questions on a given poll may have depressed stated conspiracy beliefs by triggering social desirability bias in a different way. For example, respondents may react differently when confronted with only a single or small number of conspiracy belief questions compared to a scenario where a great many are asked at once.

This task is somewhat complicated by the fact that we do not have access to the full questionnaire for every data point we employ—sometimes these were made available in the Roper Center database or by individual researchers, sometimes they were not. However, Roper does include descriptions of the topics addressed by questionnaires. We used this information to create a dummy variable coded 0 if we know or can reasonably surmise that only conspiracy belief question or a single conspiracy theory topic was addressed in a given survey, or 1 if we know that there were multiple questions about multiple conspiracy theories. We re-estimated the

model from the first column of Table A6, including this new dummy variable; results appear in the second column.

We find a significant positive impact of the number of conspiracy belief questions asked on a survey on average support for conspiracy theories (note, however, that we do not observe a significant bivariate correlation between beliefs and this variable, p=0.981). As with the findings regarding survey mode, this result provides peace of mind: surveys with many conspiracy belief questions, which are more likely to serve as T_2 data points than T_1 data points, reveal higher levels of support for conspiracy theories, on average. If T_2 is likely to be greater than T_1, on average, this would work against our principle finding of general stability over time.

Altogether, we find little reason for concern that survey mode or questionnaire design are systematically promoting the results we present in the main text. If anything, these supplemental analyses show that design effects may promote a bias in favor of an increase in conspiracy theory beliefs, rather than stability or a decrease.

Table A6: OLS regression of percentage of conspiracy believers for each conspiracy theory in Study 1, standard errors clustered by conspiracy theory question.

	1	2
Mode: Automated telephone	-10.433	-10.827
	(8.457)	(8.278)
Mode: Telephone	-11.197	3.967
	(8.586)	(9.557)
Mode: Face-to-face	-20.993	1.744
	(13.587)	(18.756)
Qualtrics?	5.265	2.671
	(8.526)	(8.438)
Year	-0.914**	-0.576
	(0.321)	(0.357)
Number of CTs on Survey		15.179*
		(7.092)
R^2	0.154	0.183
n	102	102

Note: OLS coefficients with standard errors in parentheses.
***p<0.001, **p<0.01, *p<0.05

V. Examining changes in and impact of "don't know" responses

Restricting our analysis, at first, to only those conspiracy beliefs that were polled at least 20 years before the most recent data we employ (i.e., 2001 or earlier), we are left with 10 conspiracy theories. We then computed the correlation between change in "don't know/no opinion" rates and change in beliefs (subtracting T_2 from T_1), which is –0.36 (p=0.305). Note the large p-value. This is not only because of the small number of observations, but a relatively unclear pattern, as Figure A1 below showcases. Even if the correlation were clearer and statistically significant, Studies 3 and 4 demonstrate that once the peculiarities of specific conspiracy theories have been removed from the equation, there is much more stability than change.

We also note that, when examining only the conspiracy theory questions that were first polled in the last 20 years, we still observe an average change of –2.27 percentage points in beliefs, compared to –4.57 on the full sample. In other words, our substantive conclusion—that stability is most common, and when there is change it tends to be downward—is unaffected by removal of the older polls. Indeed, of the conspiracy theory beliefs first polled during or after 2001 in Table 3, four showed a significant increase, 13 showed no change, and eight showed a significant decrease (i.e., double the number that increased).

Figure A1: Change in the percentage of conspiracy theory believers by change in the percentage of "don't know" or "no opinion" responses for T_1 polls fielded before 2001. LOWESS smoother and 95% confidence band in blue; regression line in red.

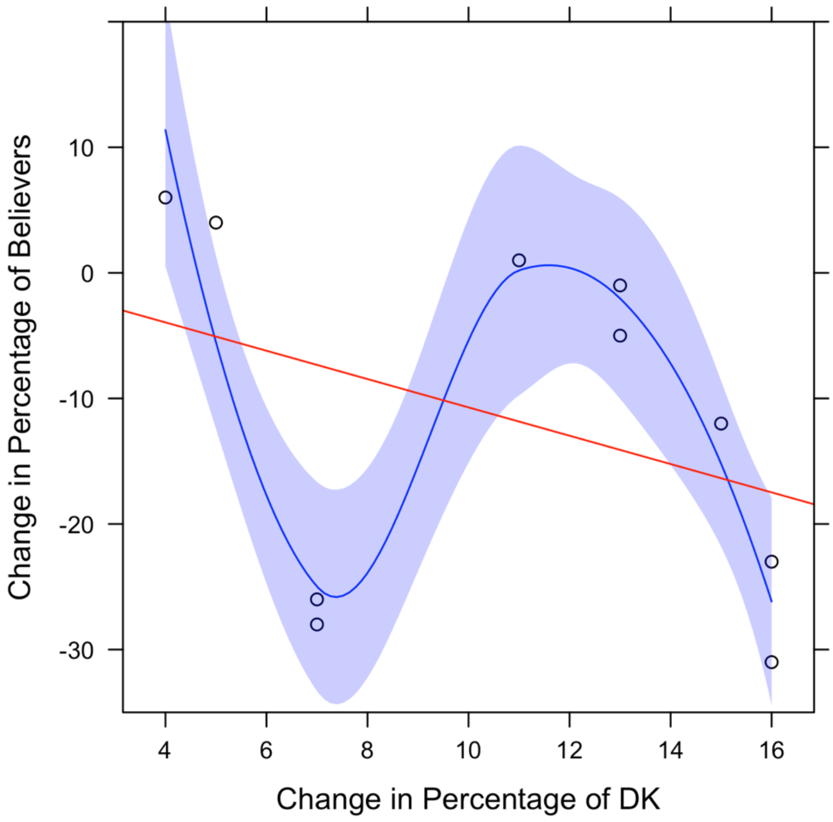

With this supplementary analysis, we wish to make two brief theoretical notes. First, it's difficult to know how "don't know" or "no opinion" should be interpreted. Many of these responses could be due to respondents not having heard of the conspiracy theory before. We don't suspect that this would stop individuals high in conspiracy thinking from providing affirmative responses—these individuals see conspiracies all around them, regardless of logic, reality, or their knowledge of the details of a given theory (robust correlations between conspiracy thinking and beliefs in specific conspiracy theories provide, some of which we present in the appendix, are highly suggestive of this). Some could have heard the conspiracy theory in question expressed in a different way or simply have no opinion, regardless of their knowledge of the event in question. Likewise, some, who might have agreed with the conspiracy theory in years prior, might now be less enthusiastic about it as positive evidence has, over decades, failed to avail itself. We have no way of distinguishing between these possibilities.

Second, an increase in "don't know" responses seems to us to be evidence against an increase in conspiracism, in anything. If people require historical details about the events involved in a given theory, then it would be hard to conclude that they have become more conspiratorial over time. Instead, it appears that (some) "old" conspiracy theories fade and new ones become relevant—this process is more consistent with replacement than a cumulative increase.

VI . Psychometric properties of the ACTS

Figure A2: Cronbach's alpha and proportion of variance accounted for by first factor of an exploratory factor analysis (iterated principal axis factoring) of the ACTS conspiracy thinking items.

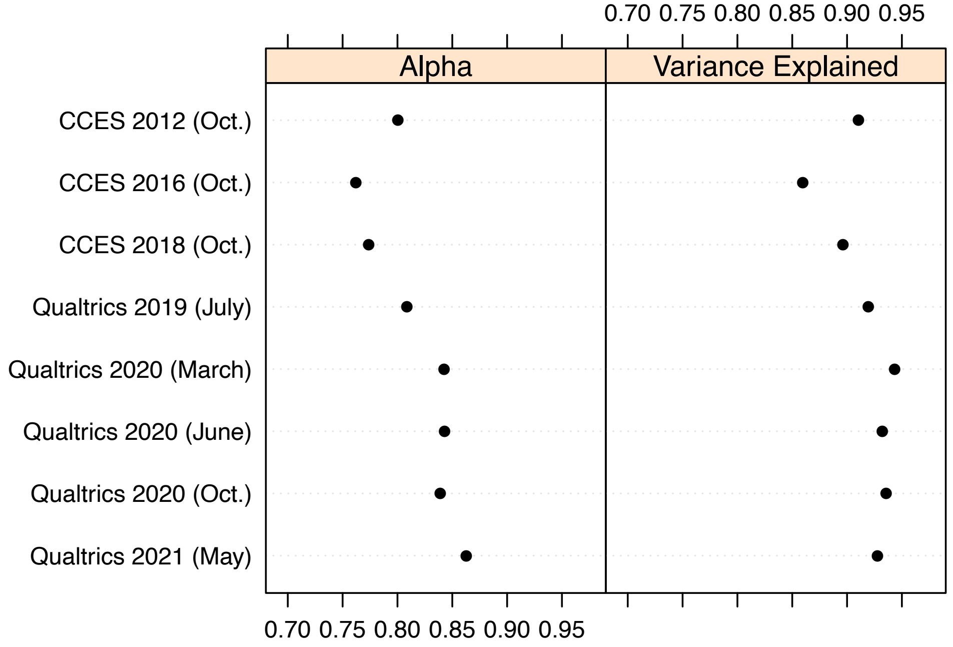

VII. Predictive validity of the ACTS

In order to showcase the validity of the ACTS, we examine the predictive power when it comes to beliefs in specific conspiracy theories. Using the May 2021 survey, we generated a variable that captures a count of all conspiracy theories endorsed by each respondent (out of 37 such beliefs on this survey). We then regressed this count on the ACTS, plus controls for partisanship, ideology, religiosity, age, educational attainment, gender, race, and ethnicity. Model results and question wording follow. Figure A3 shows the predicted number of conspiracy theory beliefs held at for people falling in lower, middle, and upper third of the conspiracy thinking scale. Those in the upper third believe more than five times the number of conspiracy theories as those in the lower third.

Question wording/variable coding:

Conspiracy thinking. (each item is 1=strongly disagree, 5=strongly agree; α=0.86, *M*=3.11, *SD*=1.00):

1. Much of our lives are being controlled by plots hatched in secret places.
2. Even though we live in a democracy, a few people will always run things anyway.
3. The people who really 'run' the country, are not known to the voters.
4. Big events like wars, the current recession, and the outcomes of elections are controlled by small groups of people who are working in secret against the rest of us.

Ideology. (self-placement; 1=extremely liberal, 7=extremely conservative)

Partisanship. (self-placement; 1=strong Democrat, 7=strong Republican)

Religiosity. “On average, I attend religious services or a place of worship: (please do not include weddings or funerals)”

1. Never
2. A few times a year
3. Once or twice a month
4. Once a week
5. Every day

Sociodemographics:

1. Educational attainment (6-point scale, 1=No high school degree, 5=post-grad degree)
2. Age (age in years, 18–96)
3. Gender (0=male, 1=female)
4. Race (Black: 0=not Black, 1=Black; White: 0=not White, 1=White)
5. Ethnicity (Hispanic: 0=not Hispanic, 1=Hispanic)

Table A7: OLS regression of count of conspiracy theory beliefs on ACTS plus controls. May 2021 survey.

Conspiracy Thinking (ACTS)	5.806***
	(0.179)
Partisanship	0.017
	(0.079)
Ideology	0.062
	(0.094)
Religiosity	0.578***
	(0.105)
Age	-0.055***
	(0.008)
Education	-0.002
	(0.092)
Female	-0.749**
	(0.264)
White	0.186
	(0.443)
Black	0.715
	(0.534)
Hispanic	0.445
	(0.501)
Constant	-2.023
	(0.784)
R^2	0.407
n	2019

Note: OLS coefficients with standard errors in parentheses.
***p<0.001, **p<0.01, *p<0.05

Figure A3: Predicted number of conspiracy theory beliefs held by level of conspiracy thinking, holding control variables at their mean.

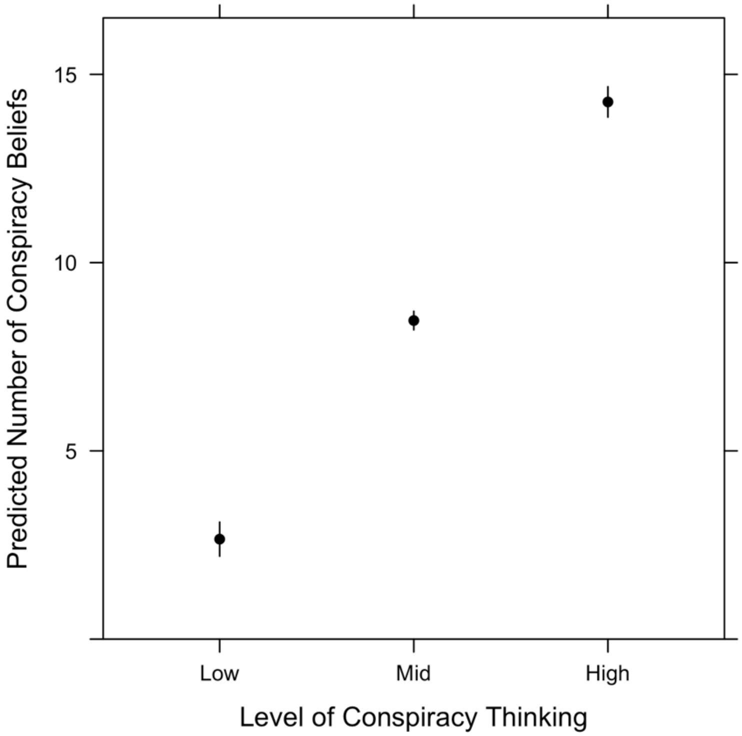

Supplement: S1 File — (PDF) [file pone.0270429.s001.pdf]
